# Clinical outcome of arthroscopic internal drainage of popliteal cysts with or without cyst wall resection

**DOI:** 10.1186/s12891-020-03453-5

**Published:** 2020-07-06

**Authors:** Chao Su, Shi-da Kuang, Xin Zhao, Yu-sheng Li, Yi-lin Xiong, Shu-guang Gao

**Affiliations:** 1 Department of Orthopaedics, Xiangya Hospital, Central South University, 87 Xiangya Road, Changsha, 410008 Hunan China; 2 Hunan Key Laboratory of Joint Degeneration and Injury, Changsha, China; 3 Hunan Engineering Research Center of Osteoarthritis, Changsha, China; 4 National Clinical Research Center of Geriatric Disorders, Xiangya Hospital, Central South University, Changsha, China

**Keywords:** Knee, Popliteal cyst, Arthroscopy, Cystectomy, Cyst wall

## Abstract

**Background:**

This study aimed to compare the arthroscopic internal drainage of popliteal cysts alone or in combination with cyst wall resection in terms of clinical outcomes.

**Methods:**

Forty-two consecutive patients with symptomatic popliteal cysts received arthroscopic treatment. Specifically, 20 of them received arthroscopic internal drainage (AI group) alone and 22 received arthroscopic internal drainage combined with cyst wall resection (AICR group) through double posteromedial portals. Magnetic resonance imaging (MRI) was performed to identify recurrence of popliteal cysts. The Lysholm score and Rauschning-Lindgren grade were used to assess the clinical outcomes. The median of the follow-up period was 24 months (12–48 months).

**Results:**

The two groups (AI group and AICR group) were similar in age, gender, cyst diameter, associated joint disorder, preoperative Lysholm score, preoperative Rauschning-Lindgren grade and follow-up period (*P* > 0.05). Relative to the AI group, the AICR group had a significantly prolonged operation time (*P* < 0.05) and a higher incidence of complications (*P* < 0.05). In both groups, the Rauschning-Lindgren grade at the last follow-up significantly differed from the preoperative grade (*P* < 0.05) and the Lysholm knee score remarkably increased compared to the preoperative score (*P* < 0.05); however, there were no differences between the two groups at the last follow-up (*P* > 0.05). According to the MRI results, the cyst disappeared in 11 (55%), shrank in size in 6 (30%) and existed in 3 (15%) patients in the AI group, and was absent in 18 (81.8%) and shrank in size in 4 (18.2%) patients in the AICR group, suggesting a significant difference between the two (*P* < 0.05).

**Conclusion:**

Additional resection of cyst wall can result in a lower recurrence rate of cysts but extend the operation time and increase the incidence of perioperative complications compared with arthroscopic internal drainage of popliteal cysts alone.

## Background

Popliteal cysts, or Baker’s cysts [1], are typically characterized by enlargement of the gastrocnemius-semi-membranosus bursa, which communicates with the knee often through a valve-like structure associated with knee osteoarthritis or meniscus tear [2]. The presence of a one-way valve leads to unidirectional flow of fluid from the articular cavity to the bursa. This is a fundamental factor that is involved in the formation and persistence of cysts [3]. The related clinical problems are closely associated with the size and location of the cyst; specifically, commonly-seen symptoms include: popliteal mass or swelling (76%), knee effusion (32%), pain (32%), thrombophlebitis (13%), buckling of the knee (11%), clicking of the knee (11%), and locking of the knee (3%) [4].

With respect to symptomatic cysts, resection has been indicated as the preferred treatment. Open excision is historically a classical method, which needs to detect the integrity of the cyst and completely resect the cyst wall; however, large wounds and high recurrence rates (42–63%) have been reported [3–6]. Recently, arthroscopic intervention is being popularized and a lot of arthroscopic treatment methods for popliteal cysts have been proposed [7–11]. Several studies have reported excellent outcomes of arthroscopic internal drainage of popliteal cysts without cystectomy by broadening the opening of cysts to eliminate the unidirectional flow [7–10]. Concomitant cystectomy which may lower the recurrence rate has yielded satisfactory therapeutic outcomes [11–13]. Compared with arthroscopic decompression of cysts alone, the additional posterior open cystectomy has demonstrated a potential to reduce the recurrence of popliteal cysts [14]. Unfortunately, studies that directly compared arthroscopic valve debridement with and without cystectomy were rarely reported for the time being.

This study aimed to compare the clinical results of arthroscopic internal drainage of popliteal cysts alone or in combination with cyst wall resection.

## Methods

**Fig. 1:**
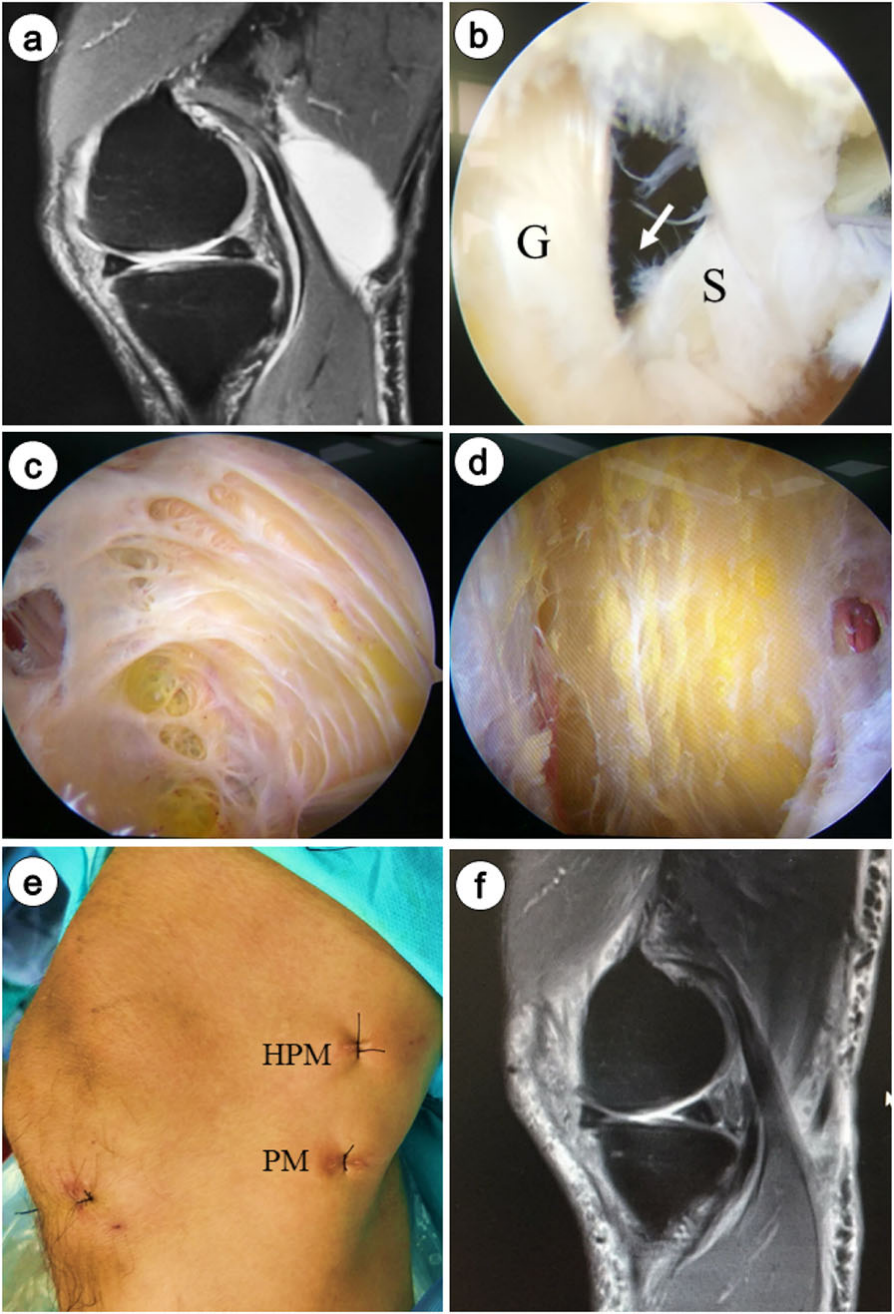
**a** Pre-operative MR images demonstrate a popliteal cyst on the right knee, **b** Arthroscopic view of the opening (white arrow) between the popliteal cyst and joint space, after we resected the capsular fold with a shaver and exposed the medial head of gastrocnemius (G) and the semimembranosus tendon (S), **c** Arthroscopic view of the inner cystic wall of the popliteal cyst via posteromedial portal, **d** Arthroscopic view after the cyst wall excision was completed, **e** Postoperative photograph showing external landmarks of posteromedial (PM) portal and high posteromedial (HPM) portal, **f** Last follow-up (24 months after surgery) MR images demonstrate no recurrence of the popliteal cyst on the right knee

With approval from Institutional Review Committee, the data of 42 patients diagnosed of symptomatic popliteal cysts who received arthroscopic treatment from January 2012 to January 2019 were reviewed retrospectively. All participants submitted the written informed consent for participation. The patients who qualified the inclusion criteria and completed ≥12 months of clinical follow-up were finally included in our study. Preoperative X-ray radiography and magnetic resonance imaging (MRI) were performed for purposes of diagnosis confirmation and patient classification. There were no asymptomatic popliteal cyst cases in this study and all of the popliteal cysts were found on the medial side. All patients were evaluated by the Kellgren-Lawrence system (KL), Lysholm score, and Rauschning-Lindgren grade [15]. The position of the popliteal cyst was determined based on pre-operative MR images (Fig. 1a), with detection of the related knee pathologies (e.g., anterior cruciate ligament lesions, chondral lesions, meniscal tears, and degenerative arthritis or synovitis) (Table 1).

**Table 1:** Patient Characteristics in the AI group and the AICR group

	AI group (*n* = 20)	AICR group (*n* = 22)	*P*-value
Age (years)	48.2 ± 9.0	49.5 ± 7.2	> 0.05
Gende (M/F)	7/13	8/14	> 0.05
Associated joint disorder			
–Synovitis	5 (25%)	6 (27.3%)	> 0.05
–Medial meniscal tear	4 (20%)	5 (22.7%)	> 0.05
–Lateral meniscal tear	2 (10%)	2 (9.1%)	> 0.05
–Chondral degeneration	12 (60%)	13 (59.1%)	> 0.05
–Loose body	1 (5%)	1 (4.5%)	> 0.05
Cyst diameter (cm)	5.56 ± 1.38	5.72 ± 1.19	> 0.05

Patients with any of the following conditions, i.e., K-L grade greater than III, knee joint instability, ligament injury, previous operation on the same knee, recurrent popliteal cyst, or history of lower extremity vascular disease, were excluded from the study. All the included patients had received non-operative treatment for at least 3 months. The indications for surgical intervention included the detection of cystic lesion on MRI accompanied by symptoms correlated with intra-articular diseases and mass-like symptoms (e.g., swelling, pain as well as restricted joint mobility).

A total of 42 patients were eventually included in our research, among which 20 received arthroscopic internal drainage (AI group) alone and 22 received arthroscopic internal drainage combined with cyst wall resection (AICR group) through double posteromedial portals. The patients diagnosed of symptomatic popliteal cysts were either treated with AI before 2017 or with AICR after January 2017. All the surgeries in both groups were performed by the same surgeon. The study group consisted of 16 men and 26 women, with an average age of 49 years (31–60 years) and a mean follow-up of 24 months (12–48 months).

### Surgical methods

#### Arthroscopic internal drainage (group AI)

General anesthesia was implemented during the operation with each patient being placed in supine position. A thigh tourniquet was typically used to control bleeding and improve visualization.

The first step was to create standard anterolateral (AL) and anteromedial (AM) portals and to carry out routine arthroscopic examination. The related intra-articular lesions, such as synovitis, chondral lesions, and meniscus tear, were then treated with the corresponding arthroscopic intervention (i.e., synovectomy, chondral lesion debridement, and meniscectomy, respectively).

**Fig. 2:**
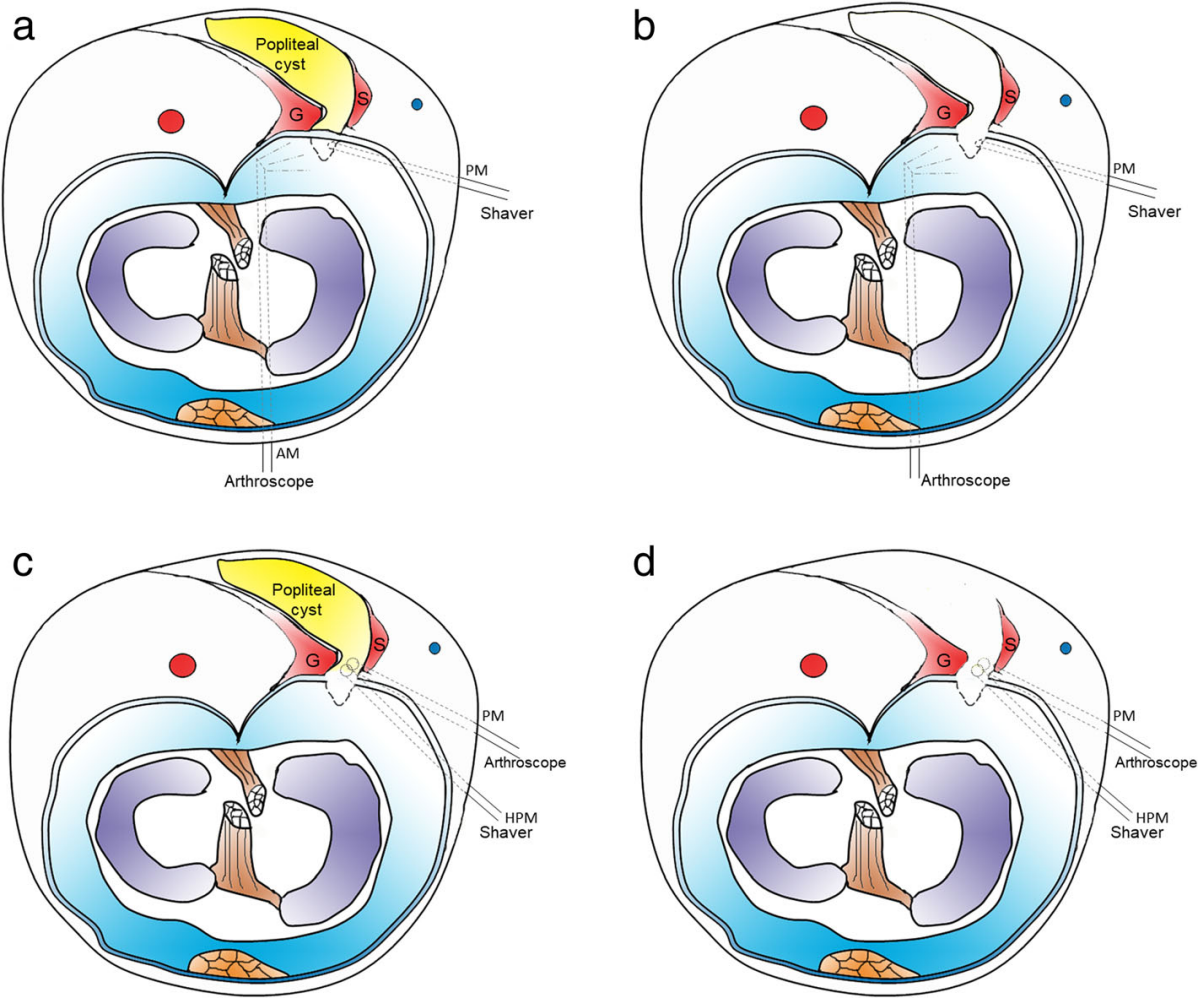
Schematic cross-section of the knee. **a** A schematic view showing the positions of the arthroscopy via the anteromedial (AM) portal and the shaver via the posteromedial (PM) portal. **b** The arthroscopy and shaver were inserted to resect the capsular fold to reestablish normal bidirectional communication and expose the medial head of gastrocnemius (G) and the semimembranosus tendon (S). **c** This drawing shows the locations of the posteromedial (PM) viewing portal and the high posteromedial (HPM) working portal. **d** The cyst wall excision was completed through the high posteromedial (HPM) working portal

The second step was to create a standard posteromedial (PM) portal. An arthroscope was introduced through the AM portal into the posteromedial compartment with 90° knee flexion, through the gap between the medial femoral condyle and the posterior cruciate ligament (PCL)(Fig. 2a). Visualization was enabled for the posteromedial compartments to identify the location of the transverse posteromedial synovial folds and the medial gastrocnemius tendon (Fig. 1b). The PM portal (Fig. 1e) was then established under the light.

The third step was to excise the capsular fold to enlarge the cyst orifice. A probe was introduced, and the communicating door to the popliteal cyst was revealed. The opening (Fig. 1b), which generally locates at the posteromedial side of the medial head of the gastrocnemius, is sheltered occasionally by a gracile and flexible membrane. A shaver was introduced and placed close to the medial head of the gastrocnemius muscle to clean the articular space. We resected the capsular fold by the shaver through the PM portal and revealed the medial head of the gastrocnemius (Fig. 2b). Basket forceps or shaver was used to enlarge the valvular opening by removing part of the capsular fold to reestablish normal bidirectional communication (Fig. 1b).

Upon accomplishment of the arthroscopic internal drainage of popliteal cyst, the articular cavity was thoroughly cleaned and flushed after hemostasis.

#### Arthroscopic internal drainage combined with cyst wall resection (group AICR)

The first three steps are the same as group AI.

The fourth step was to create a high posteromedial portal. The arthroscope was switched to the PM portal via a switching stick, and then extended into the popliteal cyst wall along the posteromedial side of the medial head of the gastrocnemius to observe the inner cyst wall. The scope was maintained in the posteromedial portal but drawn back slightly into the extra-capsular area. A #12 needle was used as a guiding probe under visualization to create a high posteromedial (HPM) portal (Fig. 1e) 3–4 cm proximal to the posteromedial portal (Fig. 2c).

The fifth step was to perform cyst wall resection. The arthroscope was inserted into the popliteal cyst wall (Fig. 1c). The septum, cyst wall and all the loose fragments were resected from the inside of the cyst by a shaver through the HPM portal (Fig. 1d). Thereafter, the scope was switched to the HPM portal via a switching rod and the PM portal was treated as the working portal. Then, the remnant cyst wall between the semimembranosus tendon and the medial head of the gastrocnemius was resected (Fig. 1d, Fig. 2d).

Upon accomplishment of the arthroscopic internal drainage combined with cyst wall resection, the articular cavity was thoroughly cleaned and flushed after hemostasis.

#### Postoperative rehabilitation

Isometric exercises for quadriceps and straight-leg raising exercises began immediately after the operation. Full weight-bearing was allowed 2 days after operation as tolerated. Active knee flexion and muscle strength exercises were allowed about 3 months after operation.

#### Clinical evaluation

At the last follow-up, MRI was performed to identify the recrudescence of popliteal cyst. The knee functions and the popliteal cysts were assessed by the Lysholm score and Rauschning-Lindgren grade, respectively.

### Statistical analysis

Statistical analyses were conducted in SPSS software version 22.0 (SPSS Inc., Chicago, IL, USA). The data was displayed as average ± standard deviation. The statistical significance of differences in continuous data, such as age, cyst diameter, Lysholm score, surgical time and follow-up period between groups, was examined by Student t test or Mann-Whitney U test in view of the assumption of normality and homoscedasticity. Differences in categorical variables (sex, affected side, associated joint disorder, Rauschning-Lindgren grade) between groups were examined by Pearson chi-square test or Fisher exact test. Student t test was used to statistically compare postoperatively and preoperatively in terms of continuous variables. A *p* value < 0.05 was considered as statistically significant.

## Results

The patient characteristics in the AI group and the AICR group are displayed in Table 1. There were no marked differences between the two groups in preoperative measurements including age, gender and cyst diameter (measured as the longest dimension in the superior-inferior length of the sagittal plane on MRI).

In the AI group, the distributions of articular pathologies associated with Baker cysts were as follows: synovitis in 5 knees (25%), medial meniscal tear in 4 knees (20%), lateral meniscal tear in 2 knees (10%), chondral degeneration in 12 knees (60%) and loose body in one knee (5%). The corresponding findings in the AICR group were: synovitis in 6 knees (27.3%), medial meniscal tear in 5 knees (22.7%), lateral meniscal tear in 2 knees (9.1%), chondral degeneration in 13 knees (59.1%) and loose body in one knee (4.5%). No marked differences were found in terms of associated joint disorder between the two groups (Table 1).

No differences were found in preoperative Rauschning-Lindgren grade, preoperative Lysholm score and follow-up period between the AI group and the AICR group (Table 2). The main complications were as follows: hematoma formation (*n* = 1 of 20, 5%) in the AI group, hematoma formation (*n* = 3 of 22, 13.6%) and extravasation under gastrocnemius muscle (n = 1 of 22, 4.5%) in the AICR group. All these complications were resolved after compression and rest. The AICR group had a longer mean operation time (57.5 ± 19.3 mins vs 39.6 ± 17.5 mins, *P* < 0.05), and a higher incidence of complications (4 cases vs 1 cases; *P* < 0.05) than the AI group did.

**Table 2:** Comparisons of data in operative time, complications, Lysholm score, Rauschning-Lindgren grade, outcome of MRI scan and mean follow-up period between the AI group and the AICR group

	AI group (*n* = 20)	AICR group (*n* = 22)	*P*-value
Operative time (min)	39.6 ± 17.5	57.5 ± 19.3	< 0.05
Complications	1	4	< 0.05
–Hematoma formation	1	3	
–Extravasation under gastrocnemius muscle	0	1	
–Poor wound healing	0	0	
–Neurovascular injury	0	0	
–Infection	0	0	
Preoperative Lysholm score	70.1 ± 10.2	71.2 ± 8.8	> 0.05
Postoperative Lysholm score	89.7 ± 6.8	90.1 ± 6.4	> 0.05
Preoperative Rauschning-Lindgren grade			> 0.05
–Grade 0	0	0	
–Grade I	2	2	
–Grade II	8	9	
–Grade III	10	11	
Postoperative Rauschning-Lindgren grade			> 0.05
–Grade 0	15	16	
–Grade I	3	4	
–Grade II	2	2	
–Grade III	1	0	
Outcome of MRI scan at the last follow-up			< 0.05
– Disappeared	11 (55%)	18 (81.8%)	
– Reduced	6 (30%)	4 (18.2%)	
– Persisted (recurrence)	3 (15%)	0 (0%)	
Mean follow-up period (months)	24.6 ± 10.5	25.2 ± 9.6	> 0.05

Rauschning-Lindgren grade: grade 0 = no pain and swelling, no range limitation; grade I = pain and swelling after intense activity, minimal range limitation; grade II, pain and swelling after normal activities, < 20 ° range limitation; grade III = pain and swelling even at rest, > 20 ° range limitation

The effects of popliteal cysts for surgical intervention were chiefly examined based on MRI at the last follow-up. The results showed that the popliteal cyst disappeared completely in 55% (11/20) of cases, shrank in size in 30% (6/20) of cases and persisted in 15% (3/20) of cases in the AI group (Table 2). Comparatively, the cyst disappeared completely (Fig. 1f) in 81.8% (18/22) of cases, shrank in size in 18.2% (4/22) of cases and persisted in 0% (0/20) of cases in the AICR group (Table 2). The difference between the two groups was significant (*P* < 0.05).

At the latest follow-up, the average Lysholm score was 89.7 ± 6.8 in the AI group and 90.1 ± 6.4 in the AICR group, suggesting no significant difference between the two (*P* > 0.05). For the final consequences of Rauschning-Lindgren grade, there were no marked differences in the distribution from grade 0 to grade 3 between the two groups (*P* > 0.05). Both groups improved significantly at the last follow-up relative to the preoperative condition (*P* < 0.05) with regard to Rauschning-Lindgren grade and the Lysholm score (Table 2).

## Discussions

Operational treatments of popliteal cysts mainly concentrate on cyst resection, the reestablishment of the bidirectional communication between the bursa and the joint cavity, and the management of intra-articular disorders. However, since the open operational resection of cysts does not concern the associated intra-articular disease, it usually results in high recurrence rates and involves a wide range of exposures and risks of vascular or neural injuries [16, 17]. In accordance with the unidirectional valve mechanism, both the reestablishment of the bidirectional connection between the joint cavity and the cyst [18] and the management of the associated intra-articular lesions play a key role to the success of popliteal cyst treatment. In recent years, arthroscopic internal drainage for popliteal cysts has been widely accepted [10, 13, 19–21].

On the other hand, open or arthroscopic closure of the cyst valvular opening has been published [22, 23]. Calvisi et al. [23] reported the all-inside arthroscopic suture technique executed in 22 patients with symptomatic popliteal cysts. These patients’ clinical outcomes were correlated with their postoperative cyst condition; no neurovascular complications were reported. The effectiveness of this technique was evaluated using MRI at 2 years postoperatively. It was found that the cyst had disappeared in 64%, shrank in size in 27%, and persisted in 9% of cases. Lindgren [24] detected the pressure in the gastrocnemius-semimembranosus bursa and the knee joint and suggested that such repairs might be inefficient for sustaining the changes of normal pressure, which could explain the relatively high recurrence rate in these communication-closure surgeries.

In our study, arthroscopic internal drainage was executed for the AI group by enlarging the one-way valve slit via two posterior portals, without resecting the inner cyst wall. The results showed that the popliteal cyst disappeared completely in 55%, shrank in size in 30% and persisted in 15% of cases over a mean follow-up of 24.6 months. Postoperative recurrence of popliteal cysts occurred in 15% (3/20) of cases in the AI group. One patient with recurrence received reoperation with AICR, and no recurrence of popliteal cyst was detected during the follow-up period. Lie and Ng published good-to-excellent results in 11 patients treated by anterior arthroscopy for an intra-articular disorder based on observation during a 13-month follow-up [25]. In their study, a posteromedial portal was created for the resection of valve slits and a trans-cystic portal was created for debriding the septum in the cases of multiple septa inside the cyst.

However, there is still controversy as to whether the arthroscopic internal drainage of the cyst alongside simultaneous management of intraarticular pathologies without cyst wall resection leads to cyst recurrence. Ko [26] retained some parts of the cystic capsule in a total of 14 cases, but they did not detect any recurrence. Shi [27] reported that concomitant cystectomy achieved no significant improvement in terms of short-term effectiveness compared with arthroscopic internal drainage alone. A recent systematic review and meta-analysis [28] compared the clinical results of arthroscopic treatments for popliteal cysts with and without cystectomy and reported satisfactory outcomes in both groups. However, the arthroscopic treatment for popliteal cysts with cystectomy was associated with a lower recurrence rate and a higher incidence of complications [28]. Our study showed that popliteal cysts could be cured by arthroscopic internal drainage with or without cyst wall resection, and the postoperative scores were both improved significantly. In the AICR group, the cyst disappeared completely in 81.8%, shrank in size in 18.2% and persisted in 0% of cases. Thus, the additional cyst wall resection could further reduce the recurrence of popliteal cysts compared with the arthroscopic decompression of cysts with treatment of intra-articular lesions alone.

The additional cyst wall resection led to a longer operation time as compared to the conventional arthroscopic internal drainage for popliteal cysts, as the AICR group required an additional high posteromedial portal and position changing. However, our study indicated that the operation time as a variable depended largely on the surgeon’s experience and on the level of difficulty of the cyst wall resection itself. Within the scope of this limited experience and the perceived learning curve (5–10 cases), improvement was felt to be achieved. Although the published literature mentioned the complications of nerve and vessel injury that might occur during the operation, the patients in our study did not exhibit such complications. Generally speaking, the complications in our study were minor, and disappeared after compression and rest. In order to avoid the complications of nerve and vessel injury during the operation, the surgeon should make sure that only the patients whose popliteal cysts are located in the medial side shall receive the operation, while the patients whose cysts are located in the outside and around the blood vessels should not be selected for the operation. At the same time, the operation must be performed gently and cautiously to avoid damaging nerves and blood vessels as far as possible. The vein crossing the posteromedial aspect of the knee joint was identified by transillumination of the arthroscopic light from the inside,thus injury to it was avoided (this in turn prevented injury to the saphenous nerve, which usually lies just posterior to the vein).Ice-compress and compression bandage were routinely used to reduce complications such as bleeding. Although there is no difference in functional outcome between the two groups, AICR can reduce recurrence. If the surgeon has mastered surgical techniques and is skilled in operation, it is meaningful to resect the popliteal cyst wall intraoperatively, which is worth popularizing.

Although our study achieved satisfactory clinical results in the management of popliteal cysts, in was subject to notable risks and limitations. The additional cyst wall resection had also been shown to introduce more potential complications, such as hematoma formation or extravasation under gastrocnemius muscle owing to the excessive use of shaver. Specifically, complications were found in 4 cases of the AICR group, and only in 1 case of the AI group, but these minor complications were all resolved after compression and rest. The popliteal artery, popliteal vein and tibial nerve also involved certain risk of injury during surgical intervention. The use of preoperative planning through MRI should help confirm whether a posterior knee cyst is a true popliteal cyst and help prevent any of these structures from injury. Several limitations of our study deserved comments. First of all, our findings were challenged by the small number of patients and retrospective reports of surgical outcomes. A limitation of the present study lies in the fact that the operations and follow-ups of different patients occurred in different time periods. Considering the current controversy over the surgical treatment of asymptomatic popliteal cysts including arthroscopy, we excluded the patients with asymptomatic popliteal cysts from our study, even though the cyst was palpable on the popliteal region and the arthroscopic treatment was executed in success. In addition, the follow-up period was generally short, and the maximum follow-up was 48 months. Further high-quality studies are needed to reassure the efficacy of operational treatment of popliteal cysts.

## Conclusions

This study demonstrated that additional resection of cyst wall could result in a lower recurrence rate of cysts but extend the operation time and slightly increase the incidence of perioperative complications compared with arthroscopic internal drainage of popliteal cysts alone. While considering the significance of the reduction in recurrence rate and ignoring the extended operation time and the slightly increased incidence of perioperative complications, an additional cyst wall resection may be a better alternative.

## Data Availability

The datasets used and analyzed during the current study are available from the corresponding authors on reasonable request.

## Abbreviations

MRI: Magnetic Resonance Imaging
AI: Arthroscopic Internal Drainage
AICR: Arthroscopic Internal Drainage Combined with Cyst Wall Resection
KL: Kellgren-Lawrence
AL: Anterolateral
AM: Anteromedial
PM: Posteromedial
PCL: Posterior Cruciate Ligament
HPM: High Posteromedial
